# Case Report: A 3’ splice site variation in *RORB* exon 3 associated with idiopathic generalized epilepsy in a child

**DOI:** 10.3389/fgene.2024.1508922

**Published:** 2025-01-17

**Authors:** Dandan Shi, Nannan Li, Caifang Fan, Qiang Luo

**Affiliations:** ^1^ Department of Pediatrics, The First Affiliated Hospital of Zhengzhou University, Zhengzhou, China; ^2^ Aegicare (Shenzhen) Technology Co. Ltd., Shenzhen, China

**Keywords:** RORB, Trio-WES, case report, epilepsy, splicing

## Abstract

The *RORB* (*Retinoic Acid Receptor-related orphan receptor β*) gene plays a crucial role in neurodevelopment and is strongly associated with bipolar disorder, cognitive function, and Alzheimer’s disease. Recently, *RORB* has also emerged as a novel potential gene involved in generalized epilepsy and absence seizures. However, due to the complexity of *RORB* gene function, reports on pathogenic variations of *RORB* genes are still lacking. In this study, we present a case of a 5-year-old epilepsy patient. Through trio whole-exome sequencing, a heterozygous variant was identified at the splice site of 3’ end of exon 3 in the *RORB* gene (chr9:77249546, NM_006914.3: c.94-1G>A). This c.94-1G>A variant disrupts normal mRNA splicing, leading to the premature termination of the RORB protein. According to ACMG guidelines, this variant is classified as “likely pathogenic”. Additionally, we provide a comprehensive summary of previously reported pathogenic or likely pathogenic variants in *RORB*, contributing to the growing body of evidence linking this gene to epilepsy. Our findings offer valuable insights into the role of *RORB* in epilepsy pathogenesis, and the splice site variant identified in this study further expands the mutational spectrum of the *RORB* gene.

## Introduction

Epilepsy is one of the most common neurological disorders, affecting up to 4% of people during their lifetime. Genetic generalized epilepsy (GGE) is a prevalent form of epilepsy characterized by seizures that originating in both hemispheres of the brain rather than being localized to a specific region. GGE typically manifests during childhood or adolescence and often exhibits a genetic predisposition within families (Rudolf et al., 2016). Among its subtypes is idiopathic generalized epilepsy-15 (EIG15), an autosomal dominant condition. The primary clinical features of EIG15 include eyelid myoclonus, seizures, developmental delay, intellectual impairment ranging from mild to severe, learning disabilities, and autism. The variability in clinical presentation among patients reflects the incomplete penetrance of the disease.

Advances in molecular genetics have enhanced our understanding of the genetic etiology of EIG15. Known genetic contributors include pathogenic variants in genes such as *SCN1A*, *SCN2A*, *GABRG2*, and *GABRA1*, among others (Hirose, 2014; Mao et al., 2023). Recent research has also suggested a potential association between mutations in the *RORB* (*Retinoic Acid Receptor-related orphan receptor β*) gene and EIG15 (Rudolf et al., 2016; Gokce-Samar et al., 2024). The *RORB* gene encodes a nuclear receptor that is highly expressed in the retina, cortex, and thalamus. It is thought to play a role in neuronal differentiation and the regulation of epilepsy (Baglietto et al., 2014; Rossini et al., 2011).

To date, there have been limited reports of pathogenic variations in the *RORB* gene in epilepsy patients. In this study, we present the case of a pediatric patient with EIG15 who carries a potentially pathogenic heterozygous variant in the *RORB* gene. We also review the relationship between *RORB* genetic variations and clinical manifestations, aiming to provide valuable genetic insights for the diagnosis and understanding of EIG15.

## Methods

### Trio whole-exome sequencing

Peripheral blood samples were collected from the affected child and both parents using EDTA anticoagulant tubes, with 2 mL of blood obtained from each individual. Genomic DNA was extracted from these samples, and Trio Whole-Exome Sequencing (Trio-WES) was conducted using the SureSelect Human All Exon V6 kit (Agilent Technologies, Santa Clara, CA) on the Illumina NovaSeq 6000 platform. The sequencing was performed at an average depth of 100× at Berry Genomics, Beijing, China. Bioinformatic analysis was applied to the sequencing data to identify potential pathogenic gene variants, which were subsequently validated through Sanger sequencing.

### ddPCR assay

To confirm whether the c.94-1G>A variant identified in the *RORB* gene exhibits mosaicism, digital droplet PCR (ddPCR) was performed on the proband and his father. This study employed the QX200™ Droplet Digital PCR System from Bio-Rad (Hercules, California, United States) and strictly followed the manufacturer’s instructions. The ddPCR reaction mixture (total volume: 20 µL) consisted of 10 µL ddPCR Supermix for Probes (No dUTP), 10 µM primers (1 µL each), 10 µM probe (1 µL), 2 µL of sample, and 5 µL of nuclease-free water (The design of primers and probes is provided in Supplementary Table S1). The prepared samples were added to a 96-well plate, centrifuged gently to ensure proper mixing, and subsequently placed into the ddPCR automated droplet generator for droplet formation. After droplet generation, the plate was sealed. The sealed PCR plate was then transferred to a T100 thermal cycler for amplification under the following thermal cycling conditions: Initial denaturation: 95°C for 10 min; 40 cycles: 94°C for 30 s (denaturation) and 55°C for 60 s (annealing); Final extension: 98°C for 10 min; Storage: 4°C.Upon completion of thermal cycling, the 96-well PCR plate was loaded into the QX200™ droplet reader for detection of each well. The resulting data were analyzed using a computer to generate the final results.

### Minigene assay

In order to analyze whether the identified mutations affect mRNA splicing, we conducted mini-gene analysis (Xu et al., 2024). The specific steps are as follows: Firstly, we constructed two types of mini-gene recombinant vectors, pcMINI-C and pcMINI (Supplementary Figure S1). Secondly, the recombinant vectors were transiently transfected into 293T and MCF-7 cell lines; transfection steps were carried out according to the liposome instructions (Rapid Plasmid Mini Kit, 1005250, SIMGEN), and samples were collected 48 h later (DNA Gel Extraction Kit, 2001250, SIMGEN) (Supplementary Table S2). Next, total RNA was extracted from the cell samples and reverse transcribed to synthesize cDNA. Finally, PCR amplification was performed using the primers pcDNA3.1-F and pcDNA3.1-R located on the mini-gene vector, and the amplified gene transcription bands were detected by agarose gel electrophoresis. Subsequently, each band was separately recovered for Sanger sequencing. The primers used are shown in Supplementary Table S3. See Supplementary Data S1 for detailed steps.

## Results

### Case report

**FIGURE 1:**
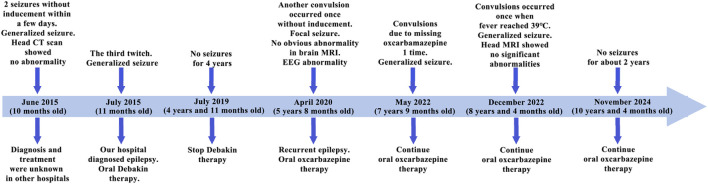
Patient history timeline. In the diagram, the light blue thick arrows represent the treatment timeline, and the dark blue arrows represent the key time points. The upper part of the arrows indicates clinical symptoms and tests, while the lower part indicates genetic diagnosis and treatment measures.

The patient is a 5-year-old female, the third child of non-consanguineous parents, born full-term without complications. Her father is healthy, while her mother has well-controlled hypertension managed with oral medication. She has two healthy older sisters, and there is no family history of epilepsy, hereditary conditions, or similar diseases. A timeline of the patient’s medical history is shown in Figure 1.

At 10 months of age, the patient experienced seizures without obvious triggers, characterized by staring, clenched fists, clenched teeth, unresponsiveness, lasting approximately 2–3 min. Upon awakening, she appeared lethargic, without fever, cyanosis, or foaming at the mouth. There is no history of infectious diseases such as hepatitis or tuberculosis, no surgical procedures, trauma, or blood transfusions, and no history of drug or food allergies. Cerebrospinal fluid examination and cranial CT were normal. After 7 days of hospitalization, she recovered and was discharged. One month after discharge, she experienced another similar episode and visited our hospital. Then, she was diagnosed with epilepsy and began oral medication (Debakin, dosage unknown). During hospitalization, there were no further seizures. After discharge, the patient continued taking medication (Debakin and levocarnitine, dosages unknown) at home with regular dose reductions until discontinuation. No seizures occurred during or after the tapering of the medication.

At 5 years of age, she experienced another seizure during sleep, again without identifiable triggers. This episode included upward eye deviation, twitching of the left eye and mouth, curling of the limbs, twitching of the left upper limb, unresponsiveness, and jaw movements starting with opening and followed by clenching. The seizures lasted about 3–4 min and resolved spontaneously. Post-seizure, she regained consciousness without symptoms such as fever, cough, headache, fainting, vomiting, or diarrhea. She subsequently returned to our hospital for treatment. Physical examination revealed that her physical development was appropriate for her age, but she demonstrated mild intellectual delay. To investigate the underlying cause, brain magnetic resonance imaging (MRI) and electroencephalography (EEG) were performed. Brain MRI was normal (Supplementary Figure S2), while EEG findings were abnormal: intermittent spikes and slow waves with moderate amplitude were observed in the right central and anterior temporal regions during wakefulness (Supplementary Figure S3). During wake-sleep transitions, scattered, paroxysmal, and continuous low-to-moderate amplitude spikes and spike-slow waves were noted in the right central, parietal, posterior temporal, bilateral occipital, and parietal midline regions, with increased frequency during sleep (Supplementary Figure S4).

Based on the clinical presentation and test results, she was diagnosed with recurrent epilepsy with focal seizures. She was prescribed oral oxcarbazepine (approximately 8 mg/kg/day), with the dosage adjusted for weight gain. During treatment, she experienced two additional seizures-one following a missed dose and another during a fever of 38°C. No further seizures have been reported since.

### Identification of a heterozygous splicing site variant in *RORB*

**FIGURE 2:**
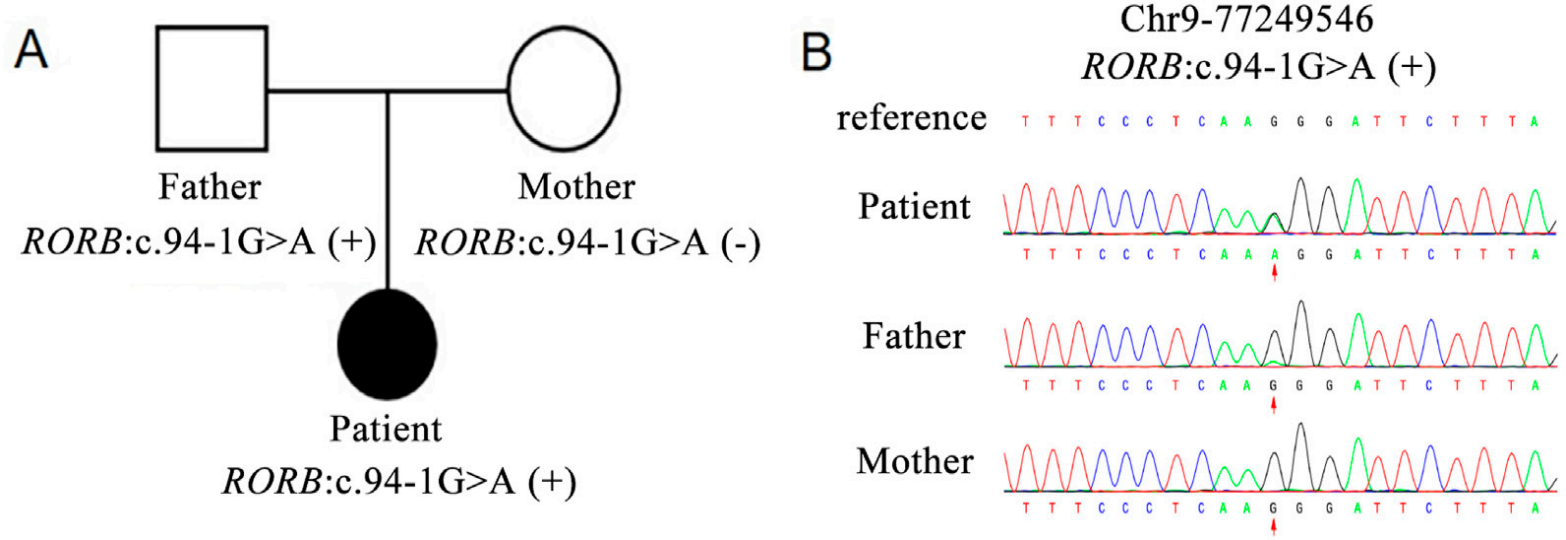
Pedigree and Sanger sequencing chromatograms of the identified variant. **(A)** Schematic presentation of the familial pedigree of the patient. White fill represents an unaffected family member, while black fill represents an affected member. **(B)** Sanger sequencing chromatogram of *RORB* in the family. The variant c.94-1G>A in *RORB* was heterozygote in the patient, mosaic in the father, and not found in the mother.

In order to further investigate the potential genetic causes of the disorder, family whole-exome sequencing was performed on the proband and her parents. The results showed that there was a heterozygous variant was identified at the splice site of 3’ end of exon 3 in the *RORB* gene, namely, chr9:77249546, NM_006914.3: c.94-1G>A, Intron2/9 (Figure 2). This variant appeared in the patient and her healthy father but not in the mother. Further, ddPCR test confirmed that the patient was heterozygote and her father was mosaic (Table 1; Supplementary Figure S5). Although this variant is recorded in the ClinVar database (Accession: VCV001683646.5), it has not been reported in the Shenzhou Genome Database, the Exome Aggregation Consortium (ExAC) database, the 1000 Genomes Project (1000G) reference population, or the Genome Aggregation Database (gnomAD), suggesting it is extremely rare. Based on these findings, we conclude that the *RORB* c.94-1G>A variant is the likely pathogenic cause of the disorder.

**TABLE 1:** ddPCR absolute quantitative detection.

Subject	Sample type	(FAM/FAAM + VIC)	Result
Patient	Blood DNA	49.20%	Positive
Father	Mouth swab DNA	23.50%	Positive
	Blood DNA	12.70%	Positive
	Hair follicle DNA	9.90%	Positive
Mother	Blood DNA	0.00%	Negative

**FIGURE 3:**
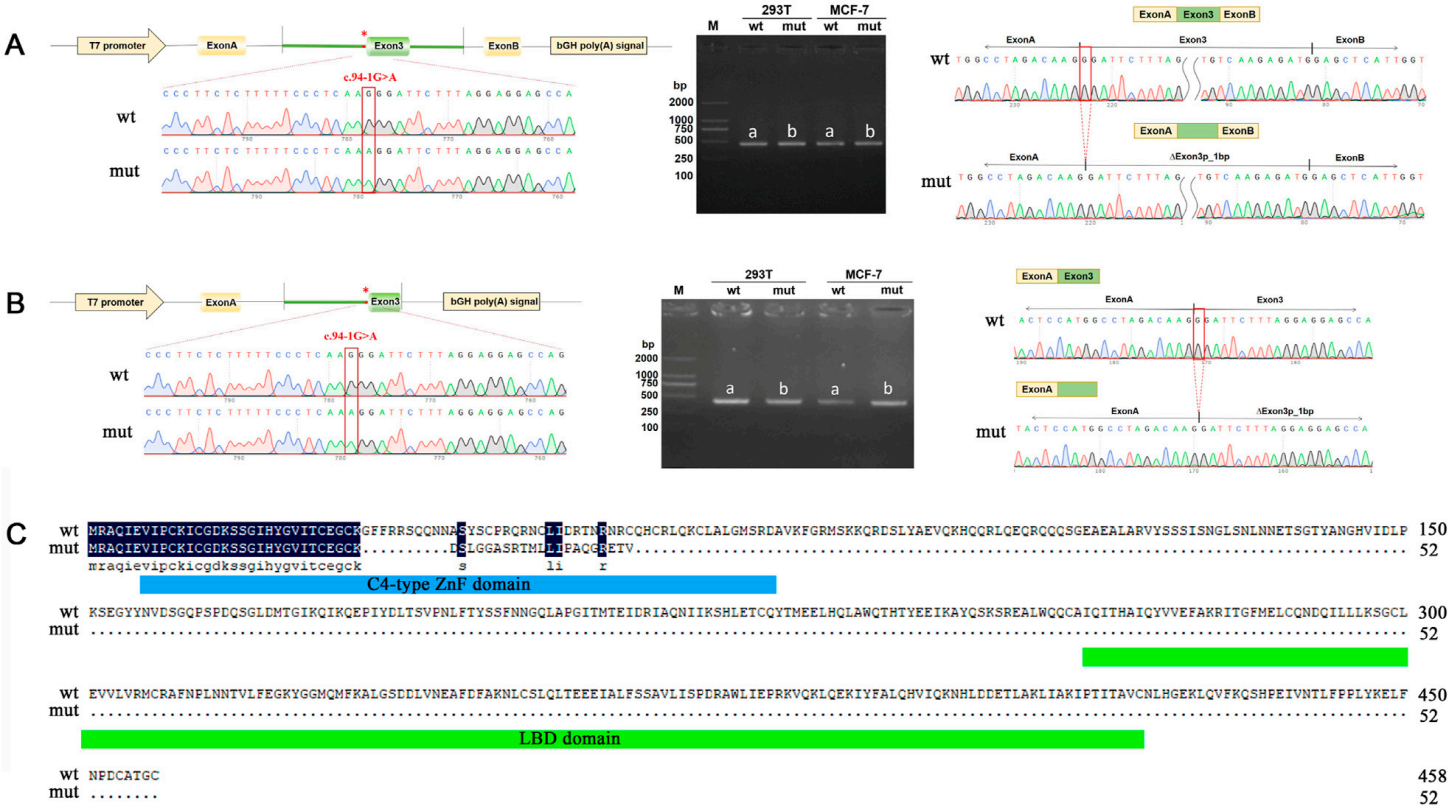
Minigene assay analysis of the *RORB* variant c.94-1G>A. **(A, B)** pcMINI **(A)** and pcMINI-C **(B)** vector used for minigene analysis. The left side shows a schematic diagram of the minigene vector construction. In the middle are the RT-PCR detection results of bands harboring spliced transcripts product in MCF-7 and 293T cells. On the right are the sequencing results of the bands harboring spliced transcripts product. a for wild type, b for mutant. **(C)** RORB amino acid sequence comparison between wild type and mutant. The blue box represents the C4-type ZnF domain and the green box represents the LBD domain (C-terminal ligand binding domain).

To verify the pathogenicity of the NM_006914.3: c.94-1G>A variant, we conducted a minigene experiment to assess its functional impact. We constructed two exon 3-expressing plasmids (pcMINI-c and pcMINI) containing the flanking sequences with and without the mutation (Supplementary Figure S1, Supplementary Data S1). These constructs were transfected into MCF-7 and 293T cells for splicing analysis. In the wild-type constructs, normal splicing patterns were observed. However, in the mutant constructs, the variant disrupted normal splicing in exon 3, leading to the deletion of 1 bp at the exon 3 (Figure 3; Supplementary Figure S6). This deletion is represented as c.94del p. Gly32Aspfs*22 at the cDNA and protein levels. The mutation introduced a premature termination codon (PTC) in exon 3 (Figure 3C), likely resulting in a truncated protein and subsequent loss of RORB function. These findings align with previous reports linking loss-of-function mutations in RORB to epilepsy (Rudolf et al., 2016).

In summary, our experimental results demonstrate that the c.94-1G>A variant in *RORB* disrupts normal mRNA splicing, introduces a premature termination codon, and leads to early termination of RORB protein synthesis, supporting its role in the disease pathology.

### Interpretation of the variants based on the ACMG guidelines

The c.94-1G>A variant of the *RORB* gene is a rare splicing variant, and its detrimental impact on splicing has been demonstrated through *in vitro* functional experiments. According to the pathogenicity classification guidelines of the American College of Medical Genetics and Genomics (ACMG), the c.94-1G>A variant is classified as a likely pathogenic variant. The pathogenicity rating (PS2+PM2+PS3 Moderate = P) was established as follows:

1) PS2 requires confirmation of the new variant through parental verification and absence of family history. The results of family exome sequencing confirmed that the c.94-1G>A variant is not a *de novo* variant in this case.
2) PM2 requires absence of the variant in normal control populations, as documented in the ESP, 1000G, or EXAC databases (or its presence at very low frequency in the case of a recessive disease). The c.94-1G>A variant is not recorded in the aforementioned databases, meeting the requirement.
3) PS3 requires strong evidence from established functional studies, either *in vitro* or *in vivo*, supporting the detrimental effect on the gene or gene product. In this case, *in vitro* functional tests have demonstrated that the c.94-1G>A variant in *RORB* causes a 1-bp deletion in Exon 3, leading to premature termination of the RORB protein.

## Discussion

*RORB* (*Retinoic Acid Receptor-related orphan receptor β*) plays a crucial role in neural development, involving in the formation of the retina, regulation of circadian rhythms, and guidance of thalamocortical axons (Jetten, 2009; Lehrer and Rheinstein, 2023). The thalamocortical circuitry plays a critical role in seizure onset (Liu et al., 2013). In humans, *RORB* is closely associated with bipolar disorder, cognitive function, and Alzheimer’s disease, indicating its pivotal role in neurocognitive processes (Lehrer and Rheinstein, 2023; McGrath et al., 2009; Ersland et al., 2012). Recently, *RORB* has also been identified as a novel potential gene involved in human neurodevelopmental disorders, including generalized epilepsy and absence seizures. Most patients with pathogenic *RORB* mutations exhibit epileptic seizures, while some present with autism spectrum disorder and severe disruption of sleep patterns without seizures (Rudolf et al., 2016; Gokce-Samar et al., 2024; Sadleir et al., 2020). It is noteworthy that knockout of the *Rorb* gene in mice leads to retinal degeneration, alterations in circadian rhythm activity, reduced anxiety-related behavior, and ataxia, without reports of spontaneous epileptic seizures (Masana et al., 2007; Andre et al., 1998). These studies suggest the complexity of the human central nervous system, which may not be fully recapitulated by mouse models, and highlight the diversity of *RORB* gene function.

In this study, we report a 5-year-old patient with epilepsy caused by a heterozygous variation in the *RORB* gene (c.94-1G>A). This variation led to a splicing variant, resulting in the deletion of 1 bp in exon 3 and the production of a prematurely terminated RORB protein. Notably, the patient’s father also harbored a mosaic variation at the same site but did not exhibit any symptoms of epilepsy. We hypothesize that the underlying reason might be related to the dosage effect of the *RORB* gene. The mosaic variation in the father had a relatively low proportion (less than 20%, Table 1), which likely minimized the disruptive impact of the abnormal protein on normal physiological functions. In contrast, the mosaic mutation in the patient accounted for 49.95% (Table 1), suggesting that the abnormal gene product or disrupted gene function constituted a much larger proportion, ultimately leading to the development of epilepsy. This finding highlights the complexity of the disease mechanism. This complex genetic mechanism also increases the difficulty of clinical treatment. The patient in this study underwent two treatment phases, divided by the discontinuation of valproic acid in July 2019 into the first and second diagnosis and treatment phases. The first treatment involved oral administration of valproic acid, with the specific dosage unknown. During the 4 years of treatment, the patient did not experience any seizures, but epilepsy recurred 9 months after discontinuing the medication. The second treatment involved oral oxcarbazepine (approximately 8 mg/kg/day), with the dosage adjusted appropriately as the patient’s weight increased. Over the 4 years of treatment, the patient experienced only two seizures: one due to missed medication and another caused by a high fever. In the past 2 years, no further seizures have occurred, indicating a certain degree of effectiveness of the treatment plan. However, the long-term efficacy of the treatment requires further follow-up observation. Additionally, whether lifelong medication is necessary remains to be evaluated.

*RORB* encodes RORβ, a ligand-dependent transcription factor, consisting of 459 amino acids with three critical functional domains (two C4-type zinc finger domains and one caboxy-terminal ligand-binding domain, LBD). The C4-type zinc finger domain (C4-ZnF) is a highly conserved DNA-binding domain in RORB, involved in the recognition ROR response elements (ROREs) that contain the consensus motif AGGTCA preceded by a 5-bp AT-rich sequence (Feng et al., 2015). The RORs bind to ROREs as a monomer to regulate the transcription of target genes (Gawlas and Stunnenberg, 2001). The LBD is a multifunctional domain in RORB, composed of 12 classical α-helices and 2 additional α-helices, playing roles in ligand binding, nuclear localization, receptor dimerization, and interaction with coactivators and corepressors (Stehlin et al., 2001).

**FIGURE 4:**
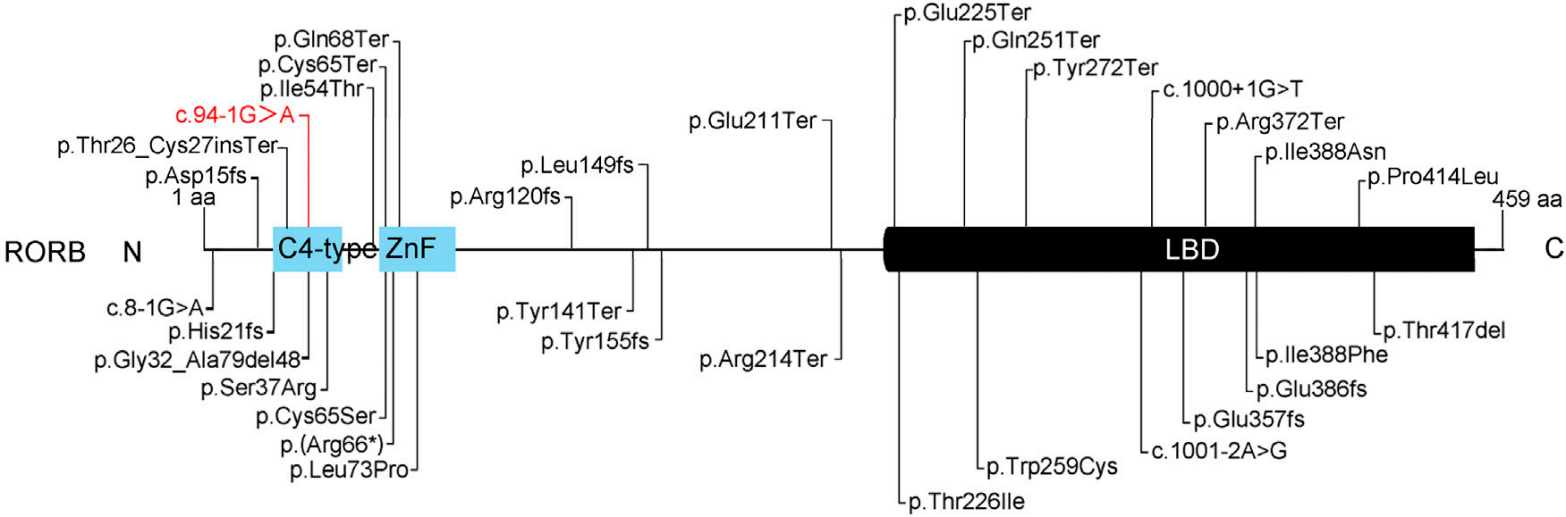
Summary of the pathogenic or likely pathogenic variants of RORB. There were 33 cases of pathogenic or likely pathogenic *RORB* gene variations, 11 in the C4-ZnF domain, 14 in the LBD domain, and 9 in other non-functional domains. Red indicates the likely pathogenic variant identified in this study.

To date, 32 pathogenic or likely pathogenic *RORB* gene mutations have been reported, along with a new potentially pathogenic variant reported in this study, totaling 33 (Figure 4). These variations are mostly associated with epilepsy, predominantly EIG15-type epilepsy. Among these variations, 10 are located in the C4-ZnF domain, 14 in the LBD domain, and 9 in other non-functional domains. Interestingly, all 9 variations in these non-functional domains are premature termination or frameshift variations, resulting in the loss of function in the C4-ZnF or LBD domains. This suggests the importance of these two functional domains in *RORB* gene function. Previous studies have shown that patients with mutations in the C4-ZnF domain of RORB exhibit more severe phenotypes compared to patients with deletions or truncations in other regions of RORB (Rudolf et al., 2016), implying that the C4-ZnF domain may be the core functional domain of RORB. The variation reported in this study is also located in the C4-ZnF domain of RORB, further indicating its pathogenicity. However, due to the limitation of experimental conditions and funding, the *in vitro* functional verification of this study is relatively lacking, which is a limitation.

## Conclusion

In this study, we report a case of 5-year-old patient with epilepsy. Through trio whole-exome sequencing, a heterozygous variant was identified at the splice site of 3’ end of exon 3 in the *RORB* gene (chr9:77249546, NM_006914.3: c.94-1G>A). This c.94-1G>A variant in *RORB* disrupts normal mRNA splicing of mRNA and leads to premature termination of the RORB protein. According to ACMG guidelines, this variant is classified as likely pathogenic variant. Additionally, we provide a summary of pathogenic or likely pathogenic variants in *RORB*. Our findings offer valuable insights into the function of *RORB* and its association with epilepsy.

## Data Availability

The original contributions presented in the study are included in the article/Supplementary Material, further inquiries can be directed to the corresponding author.
