# Supplementary material for: Case Report: A 3’ splice site variation in RORB exon 3 associated with idiopathic generalized epilepsy in a child
Source: Front Genet. 2025 Jan 17;15:1508922. doi: 10.3389/fgene.2024.1508922 (PMC11782219; doi:10.3389/fgene.2024.1508922)
Supplement: Supplementary file 1 [file DataSheet2.docx]

**Construction of minigene analysis vector for RORB gene splicing site variation**

**Recombinant Vector Construction**

Recombinant vector construction includes restriction enzyme digestion, ligation, transformation, and verification of recombinant clones. The 37°C digestion reaction (2 hours, 30 µL) contains the following components: 3 µL 10× NEB buffer, 0.6 µL of Enzyme 1, 0.6 µL of Enzyme 2, 500 ng/25 µL of vector/DNA fragment, and ddH₂O to a final volume of 30 µL. The ligation reaction (10 µL) consists of the following: 1 µL 10× ligase buffer, 7 µL of the digested DNA fragment (wt/mut), 1 µL of the digested vector, and 1 µL of ligase. After overnight ligation at 4°C, the ligated product is transformed into DH5α competent cells. The cells are cultured overnight at 37°C, and several individual colonies are randomly selected for verification. Verification methods include colony/PCR analysis and Sanger sequencing.

**Minigene Transcription Analysis**

Total RNA is extracted from cell samples using the method specified in the kit manual (see Supplementary Table 2). After measuring RNA concentration, equal amounts of RNA are reverse transcribed to synthesize cDNA. For amplification of pcMINI-wt/mut, PCR is performed using the primers pcMINI-F/pcMINI-R. For amplification of pcMINI-C-wt/mut, PCR is performed using the primers pcMINI-C-F/pcMINI-C-R (primer sequences are provided in Supplementary Table S3). The successful wild-type and mutant minigene plasmids inserted into the corresponding vectors are transfected into cells for 48 hours, followed by RNA extraction and cDNA synthesis.

**Minigene Construction**

The wild-type and mutant minigenes are inserted into the pcMINI and pcMINI-C vectors, respectively. The pcMINI-RORB-wt/mut minigene contains a portion of Intron 2 (525 bp) - Exon 3 (142 bp) - a portion of Intron 3 (480 bp), inserted into the pcMINI vector, which includes a universal ExonA-IntronA-MCS-IntronB-ExonB sequence. The pcMINI-C-RORB-wt/mut minigene contains a portion of Intron 2 (512 bp) - Exon 3 (142 bp), inserted into the pcMINI-C vector, which also contains a universal ExonA-IntronA-MCS sequence. Four recombinant vectors are transfected into MCF-7 and 293T cell lines. After 48 hours of transfection, a total of 8 RNA samples are collected. The splicing patterns of ExonA-Exon3-ExonB or ExonA-Exon3 are analyzed to determine whether any abnormalities exist (vector maps are shown in Supplementary Figure S1).
