# Supplementary material for: Case Report: A 3’ splice site variation in RORB exon 3 associated with idiopathic generalized epilepsy in a child
Source: Front Genet. 2025 Jan 17;15:1508922. doi: 10.3389/fgene.2024.1508922 (PMC11782219; doi:10.3389/fgene.2024.1508922)
Supplement: Supplementary file 2 [file DataSheet1.docx]

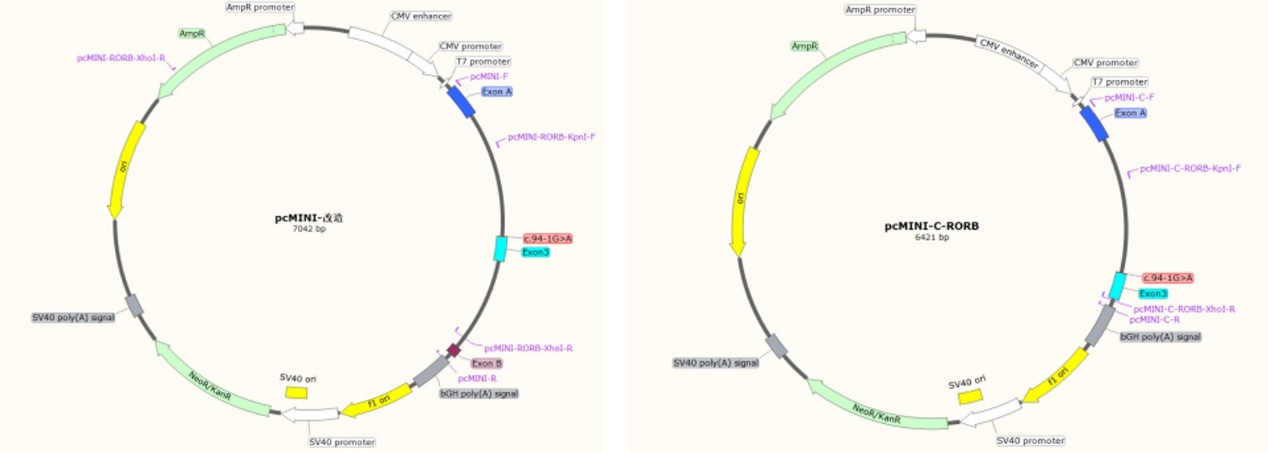


**Figure S1** **pcMINI (left) and pcMINI-C (right) vector maps.** pcMINI-c and pcMINI are modified versions of the pcDNA3.1+ vector, with improved splicing efficiency. The exons A and B on the vector are intrinsic to the vector (pcMINI-c contains only exon A; pcMINI contains both exons A and B, with higher splicing efficiency), and their sequences are derived from the ASL gene (NC_000007.14:66075819-66093576).


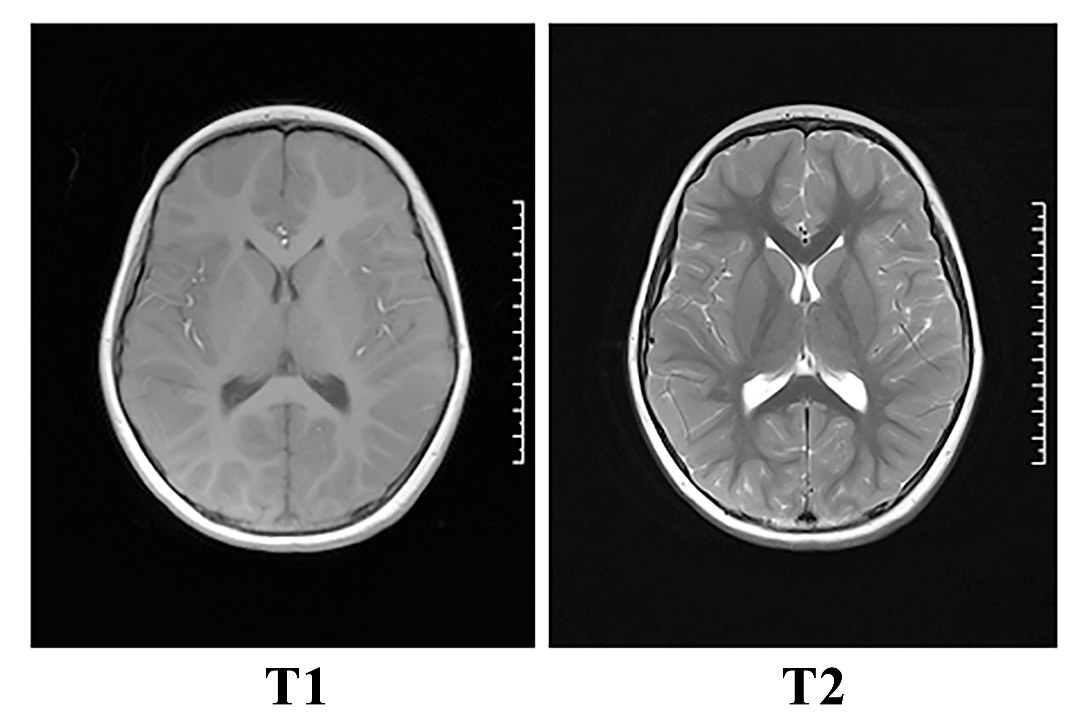


**Figure S2 Cranial MRI plain scan and high-resolution 3D-FLAIR sequence showed no significant abnormalities.**


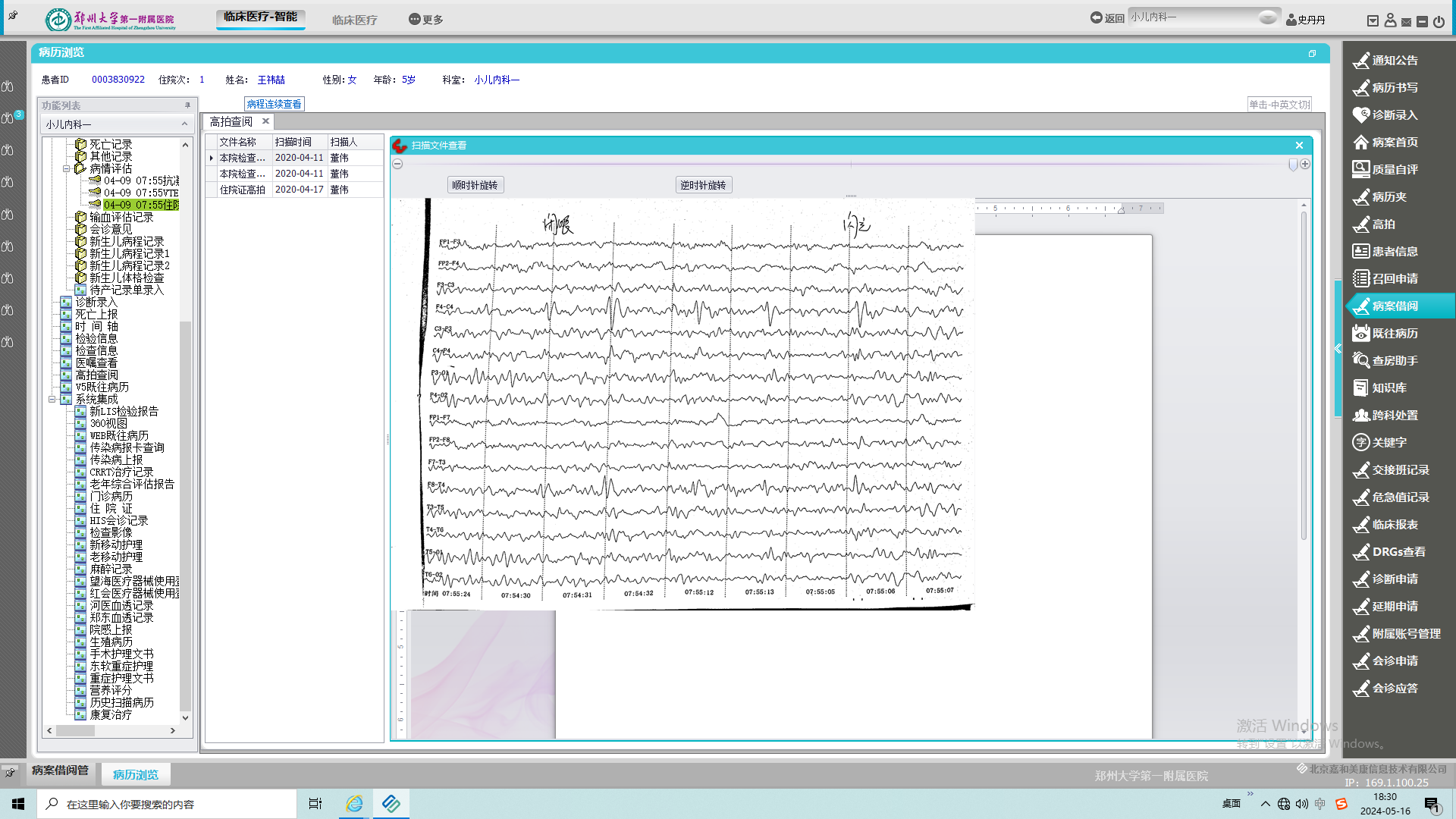


**Figure S3 Routine EEG.** Intermittent moderate-amplitude spike-and-slow wave discharges were observed in the right central and anterior temporal regions during wakefulness.


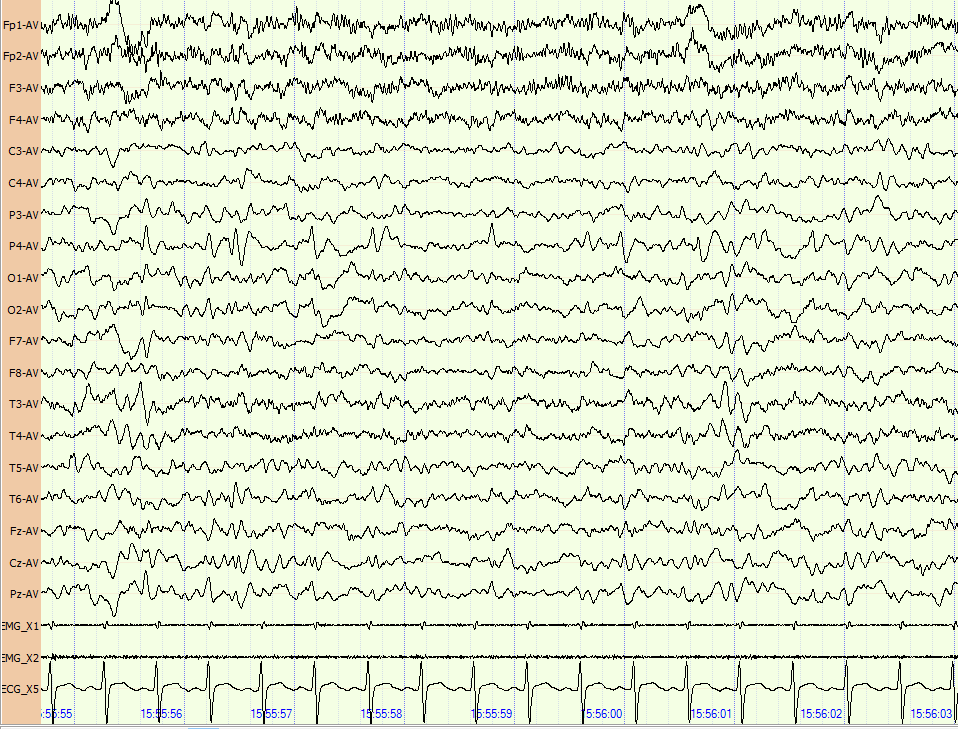

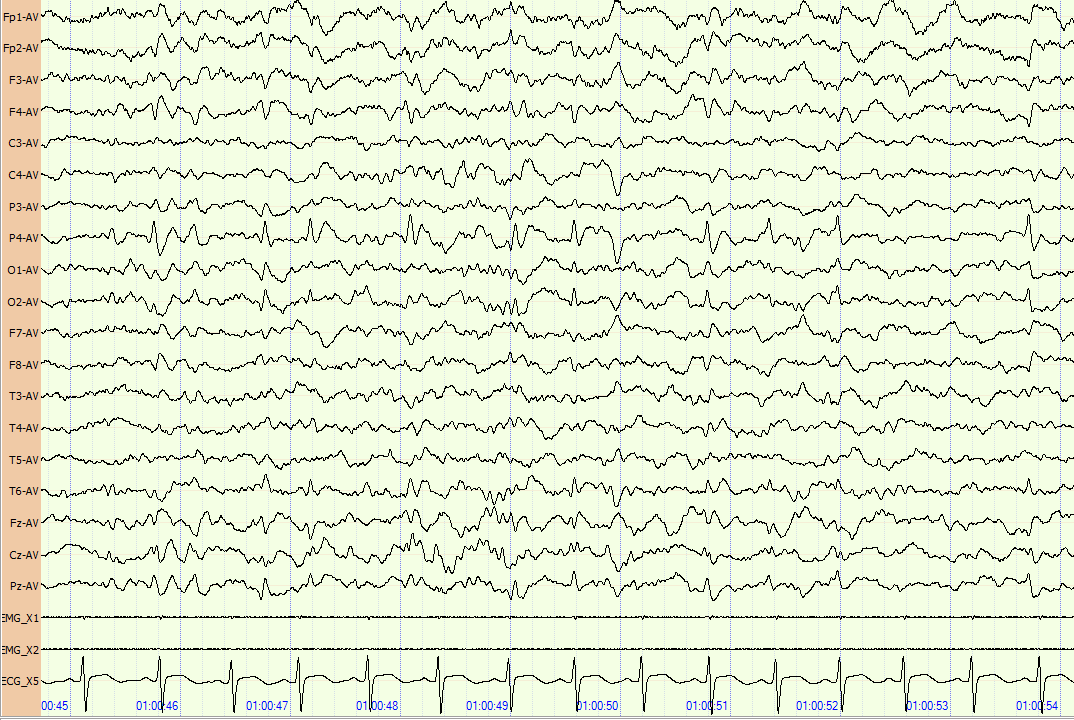


**Figure S4 Long-term EEG.** During both wakefulness (left) and sleep (right), scattered, paroxysmal, and continuous low-to-moderate amplitude spike waves and spike-and-slow wave discharges were observed in the right central, parietal, posterior temporal, bilateral occipital, and parietal midline regions, with an increased frequency during sleep. AV: Average reference lead; Fp1: Left frontal pole; Fp2: Right frontal pole; F3: Left frontal lobe; F4: Right frontal lobe; C3: Left central region; C4: Right central region; P3: Left parietal lobe; P4: Right parietal lobe; O1: Left occipital lobe; O2: Right occipital lobe; F7: Left anterior temporal lobe; F8: Right anterior temporal lobe; T3: Left mid-temporal lobe; T4: Right mid-temporal lobe; T5: Left posterior temporal lobe; T6: Right posterior temporal lobe; Fz: Mid-frontal point; Cz: Central point; Pz: Vertex; EMG-X1: Left upper limb electromyography lead; EMG-X2: Right upper limb electromyography lead; ECG-X5: Electrocardiogram lead.


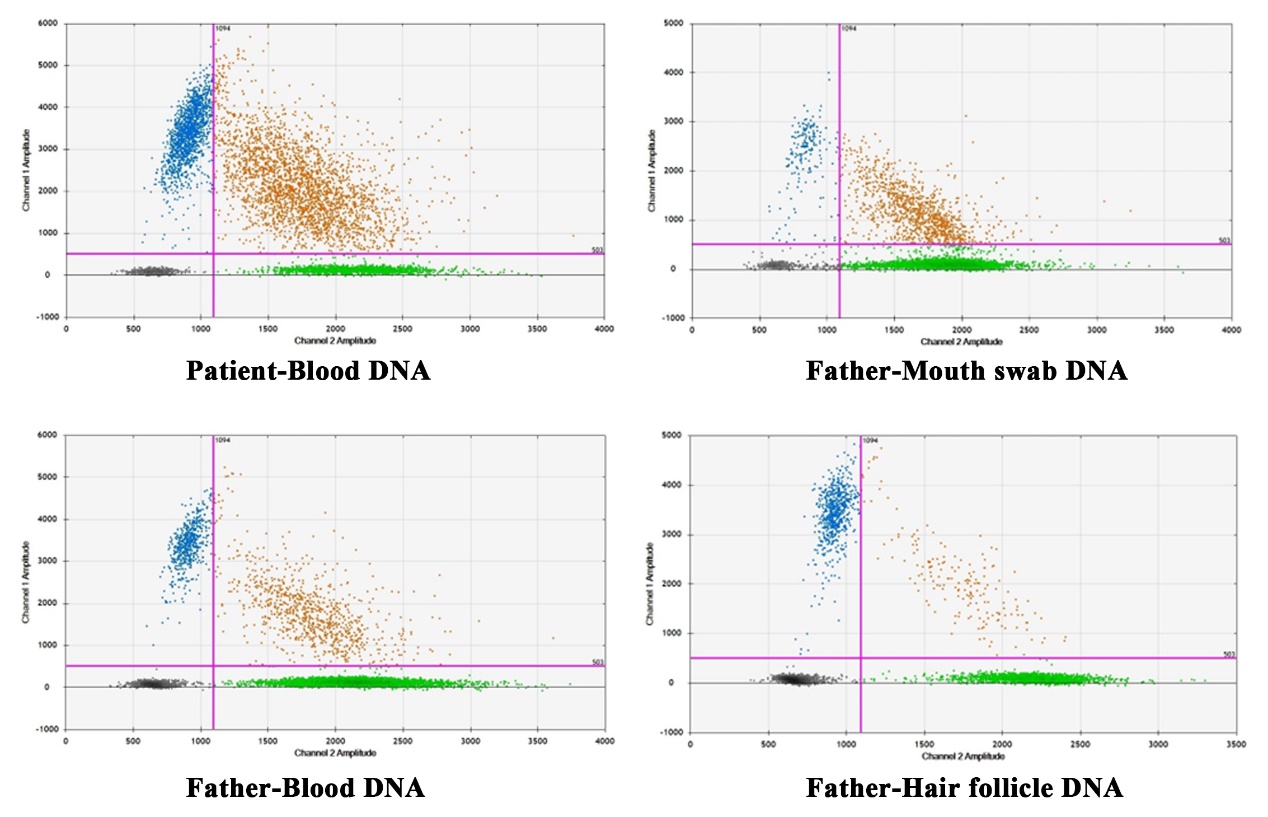


**Figure S5 ddPCR detection.** The X axis represents the channel 2 (VIC) amplitude. The Y axis represents the channel 1 (FAM) amplitude. Black indicates negative droplets, green indicates positive droplets (VIC), blue indicates positive droplets (FAM), and orange indicates double positive droplets (FAM+VIC).

**
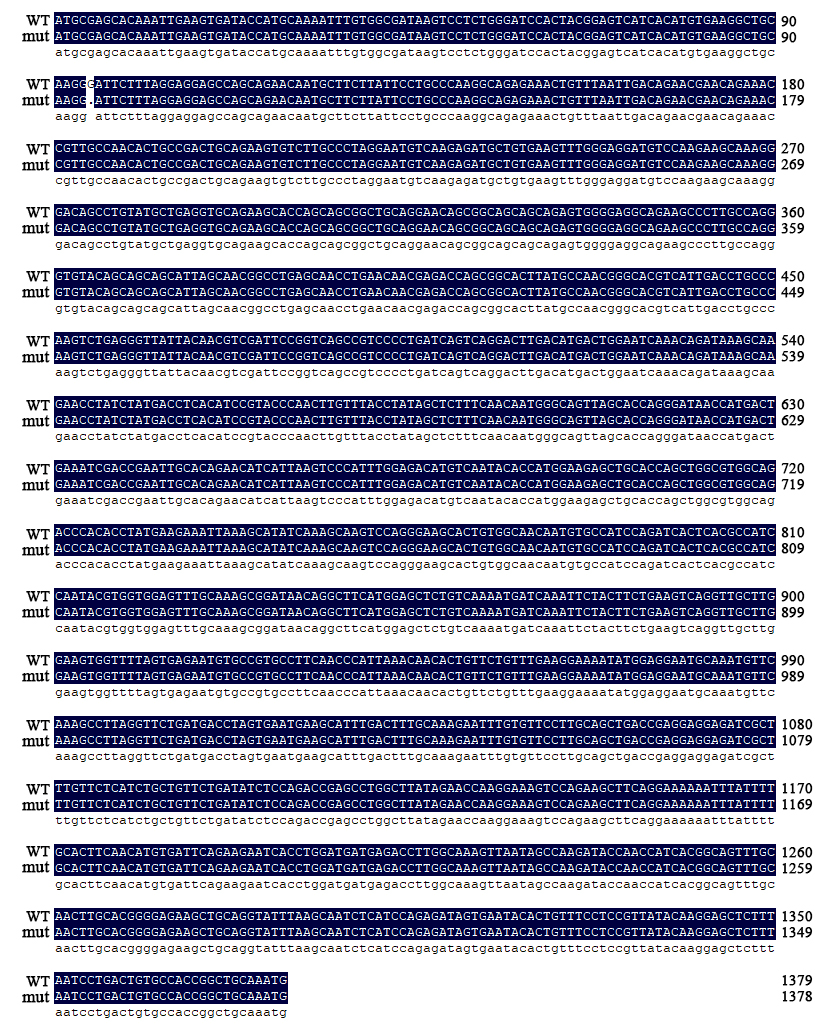
**

**Figure S6 Comparison of CDS sequences of *RORB* between wild type and mutant.**

**Table S1 List of ddPCR primers.**

| Primer name | Primer sequence |
| --- | --- |
| RORB-G77249546A-FAM | FAM-CCTCAAAGGATTCTT-MGB |
| RORB-G77249546A-VIC | VIC-CCTCAAGGGATTCTT-MGB |
| RORB-G77249546A-F | TCTGTTTCCCTCCCCTTCTCT |
| RORB-G77249546A-R | CTCTGCCTTGGGCAGGAATA |

**Table S2 Minigene test kit.**

| Product name | Article number |
| --- | --- |
| Primer STAR MAX DNA Polymerase | R045A |
| Rapid Plasmid Mini Kit | 1005250 |
| DNA Gel Extraction Kit | 2001250 |
| Trizol (RNAiso PLUS) | 9109 |
| HifairTM 1st Strand cDNA Synthesis SuperMix for qPCR (gDNA digester plus) | 11123ES70 |

**Table S3 List of Minigene primers.**

| Primer name | Primer sequence |
| --- | --- |
| 135534-F | CTCATGACACATTGCACTTT |
| 135821-F | TAGCCTGAGGCTAAGTAGAG |
| 137575-R | GTGATGTGTCCAATGTCAGC |
| 137802-R | GAATTTCACTAGAGGGAGTG |
| pcMINI-RORB-KpnI-F | GGTAGGTACCTGCATTCGAGCCAAATTCAA |
| RORB-mut-F | CTCTTTTTCCCTCAAAGGATTCTTTAGGAGG |
| RORB-mut-R | CCTCCTAAAGAATCCTTTGAGGGAAAAAGAG |
| pcMINI-RORB-XhoI-R | TTTCCTCGAGGCTATGACCAGCCAGCAGGA |
| pcMINI-C-RORB-KpnI-F | GGTAGGTACCAATTCAAAAATTTTATGGTG |
| pcMINI-C-RORB-XhoI-R | TAGACTCGAGCATCTCTTGACATTCCTAGG |
| pcMINI-F | ACTTAAGCTTATGAGTGGGCTTTGGGGTGGCCGGTT |
| pcMINI-R | TAGAAGGCACAGTCGAGG |
| pcMINI-C-F | ACTTAAGCTTATGAGTGGGCTTTGGGGTGGCCGGTT |
| pcMINI-C-R | TAGAAGGCACAGTCGAGG |

**Table S4 Full text exists for a list of abbreviations.**

| Abbreviation | Full name |
| --- | --- |
| GGE | Genetic generalized epilepsy |
| EIG15 | idiopathic generalized epilepsy-15 |
| SCN1A | SODIUM VOLTAGE-GATED CHANNEL, ALPHA SUBUNIT 1 |
| SCN2A | SODIUM VOLTAGE-GATED CHANNEL, ALPHA SUBUNIT 2 |
| GABRG2 | GAMMA-AMINOBUTYRIC ACID RECEPTOR, GAMMA-2 |
| GABRA1 | GAMMA-AMINOBUTYRIC ACID RECEPTOR, ALPHA-1 |
| RORB | Retinoic Acid Receptor-related orphan receptor β |
| EDTA | Ethylene Diamine Tetraacetic Acid |
| Trio-WES | Trio whole-exome sequencing |
| MRI | magnetic resonance imaging |
| EEG | electroencephalography |
| ExAC | the Human Exome Database |
| 1000G | the reference population of the 1000 Genomes Project |
| gnomAD | the Population Genome Mutation Frequency Database |
| ACMG | the American College of Medical Genetics and Genomics |
| ESP | Exome Sequencing Project |
| LBD | ligand-binding domain |
| C4-ZnF | The C4-type zinc finger domain |
| ROREs | the recognition ROR response elements |
